# Supplementary material for: Diazotroph Paenibacillus triticisoli BJ-18 Drives the Variation in Bacterial, Diazotrophic and Fungal Communities in the Rhizosphere and Root/Shoot Endosphere of Maize
Source: Int J Mol Sci. 2021 Feb 2;22(3):1460. doi: 10.3390/ijms22031460 (PMC7867140; doi:10.3390/ijms22031460)
Supplement: Supplementary file 1 [file ijms-22-01460-s001.pdf]

Supplemental Information

Diazotroph *Paenibacillus triticisoli* BJ-18 drives variation of the bacterial, diazotrophic and fungal communities in the rhizosphere and root/shoot endosphere of maize

Yongbin Li[1#], Qin Li [1#], Sanfeng Chen [1,*]

[1]State Key Laboratory for Agrobiotechnology and College of Biological Sciences, China Agricultural University, Beijing, 100094, P. R. China;

ybli@cau.edu.cn (Y.B.L.); lqliqin1@126.com (Q.L.); chensf@cau.edu.cn (S.F.C.)

# These authors contributed equally to this work

*Correspondence: chensf@cau.edu.cn; Tel.: +86-10-62731551

Received: date; Accepted: date; Published: date

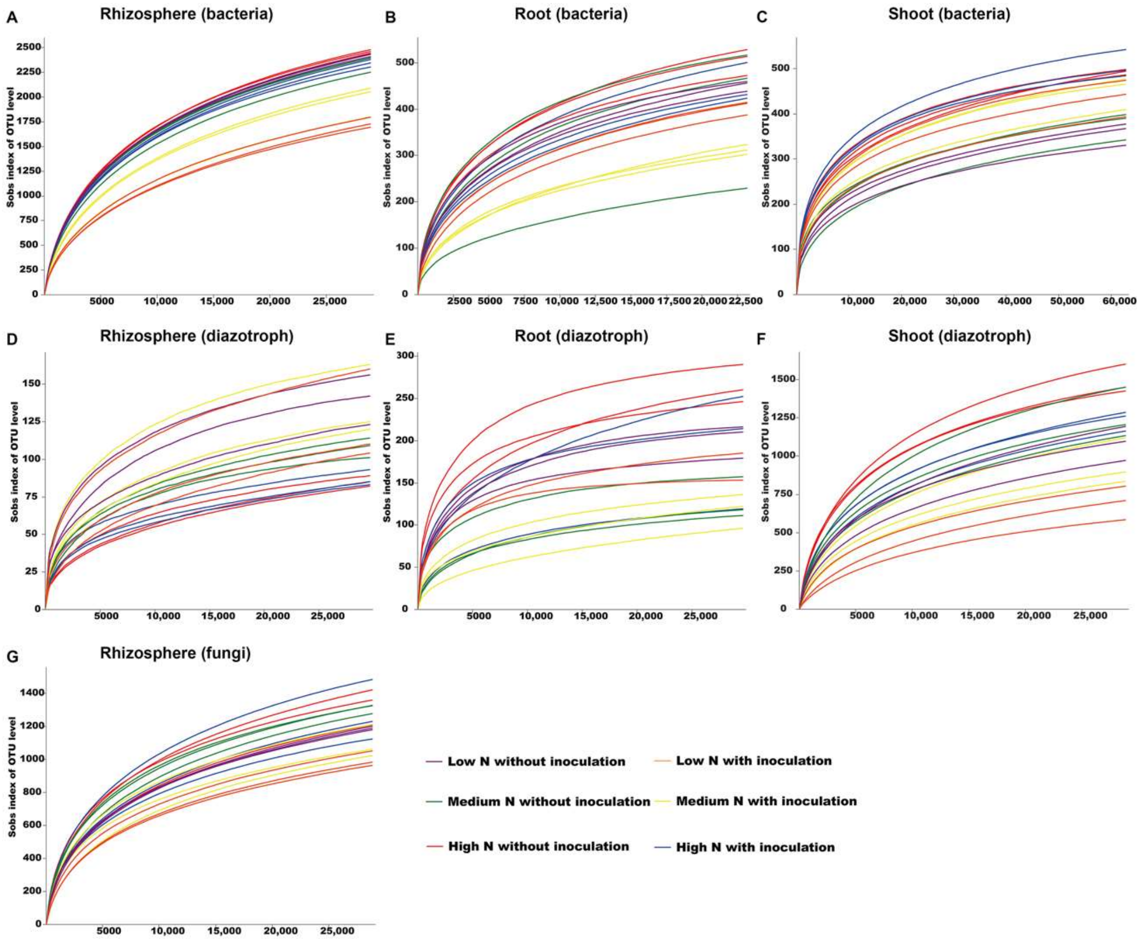

Fig. S1: The rarefaction curves of the bacterial, diazotrophic and fungal communities in the maize rhizosphere, root endosphere and shoot endosphere. A: the rhizosphere bacteria; B: the root endophytic bacteria; C: the shoot endophytic bacteria; D: the rhizosphere diazotrophs; E the root endophytic diazotrophs; F: the shoot endophytic diazotrophs; G: the rhizosphere fungi. OTUs: operational taxonomic units (97% similarity).

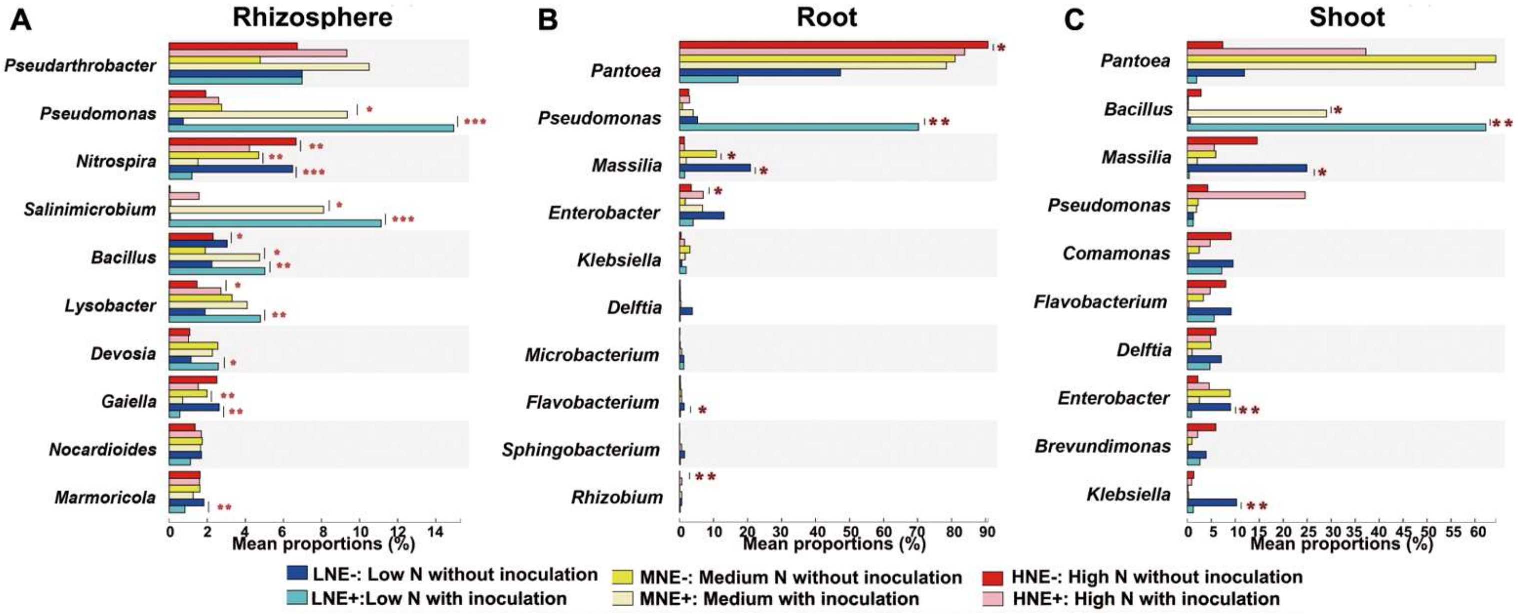

Fig. S2: The statistical comparison of the relative abundance of bacteria in the rhizosphere (A), roots (C) and shoots (E) at the genus level. The values are given as means of three independent biological replicates. Asterisk(s) (* or ** or ***) indicate significant differences between inoculated (E+) and uninoculated (E−) groups determined by Student's T at $p < 0.05$ or $p < 0.01$ or $p < 0.001$.

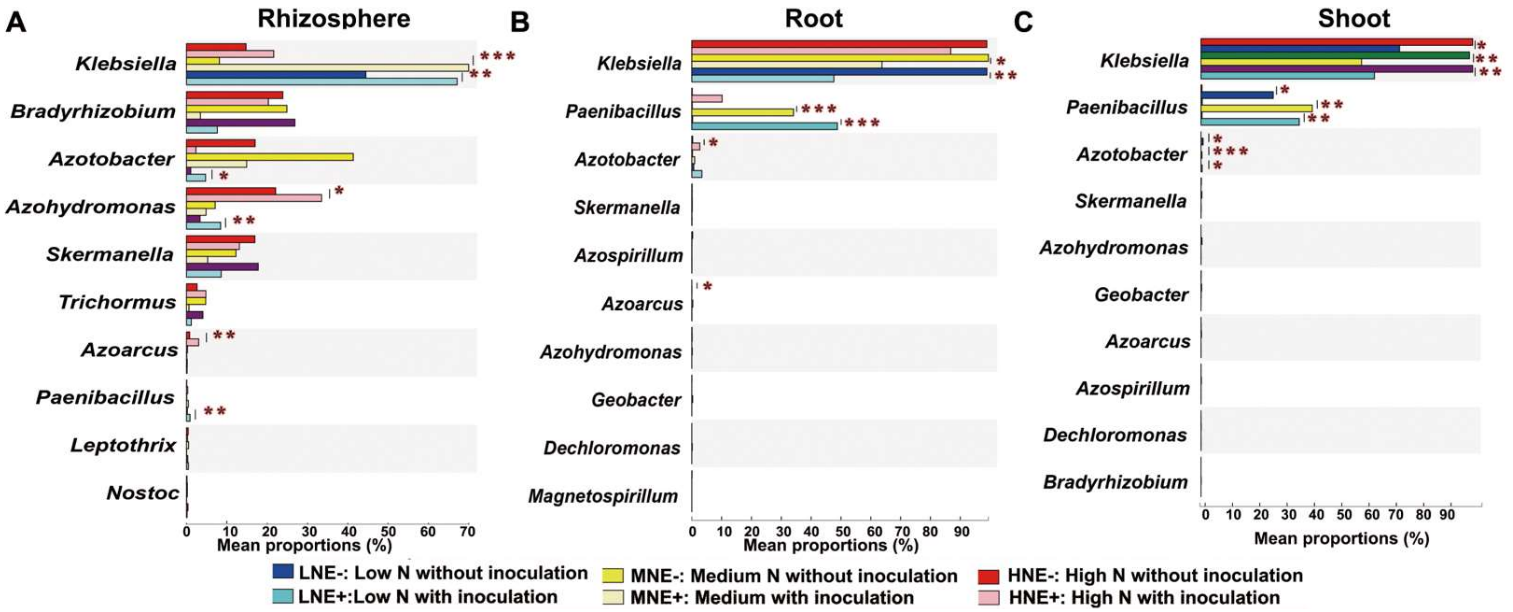

Fig. S3: The statistical comparison of the relative abundance of diazotroph in the rhizosphere (A), roots (B) and shoots (C) at the genus level. The values are given as means of three independent biological replicates. Asterisk(s) (* or ** or ***) indicate significant differences between inoculated (E+) and uninoculated (E−) groups determined by Student's T at $p < 0.05$ or $p < 0.01$ or $p < 0.001$.

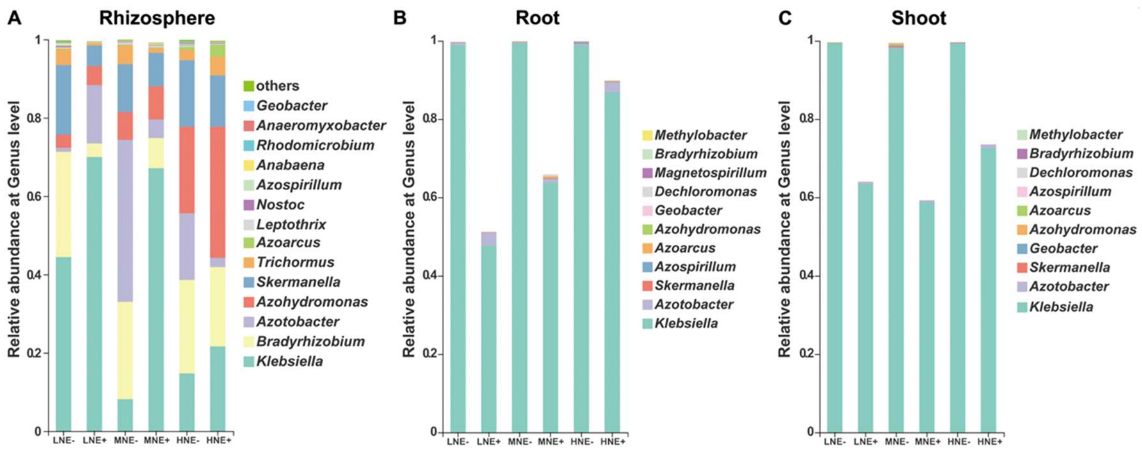

Fig. S4: The composition of the diazotrophic communities in the rhizosphere (A), root endosphere (B) and shoot endosphere (C) at genus level. LNE–: low nitrogen without inoculation; LNE+: low nitrogen with inoculation; MNE–: moderate nitrogen without inoculation; MNE+: moderate nitrogen with inoculation; HNE–: high nitrogen without inoculation; HNE+: high nitrogen with inoculation.

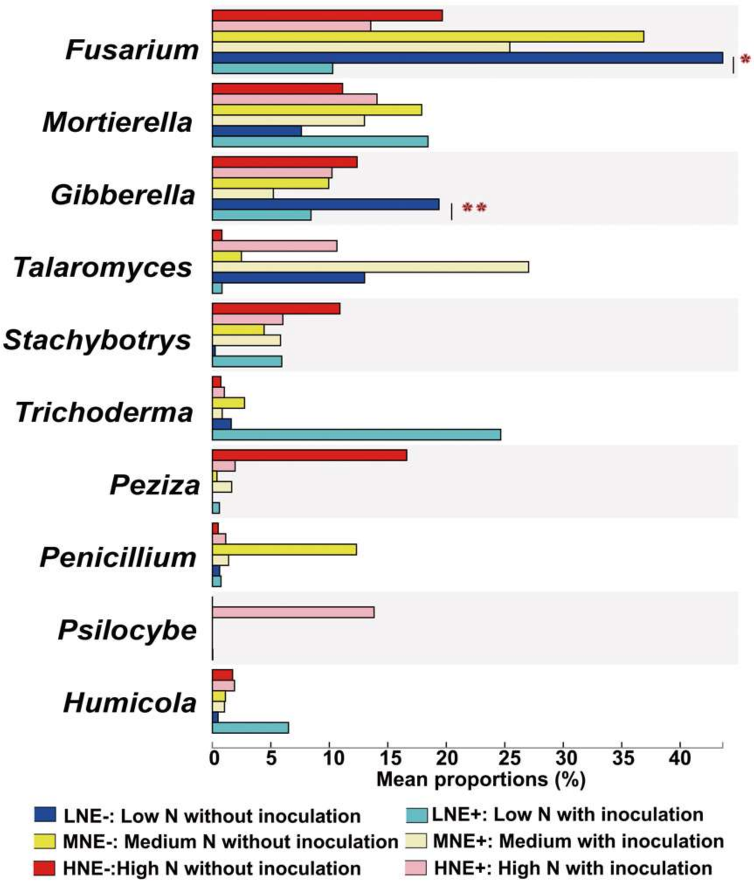

Fig. S5: The statistical comparison of the relative abundance of fungi in the rhizosphere at the genus level. The values are given as means of three independent biological replicates. Asterisk(s) (* or **) indicate significant differences between inoculated (E+) and uninoculated (E−) groups determined by Student's T at $p < 0.05$ or $p < 0.01$.

TABLE S1. Sequences of oligonucleotide primers for quantitative PCR

Target group	Primer name and sequence (5´-3´)	Size (bp) of product
16S rRNA gene	Eub338 F, ACTCCTACGGGAGGCAGCAG<br>Eub518 R, ATTACCGCGGCTGCTGG	200
ITS	ITS1 F, TCCGTA GGTGAACCTGCGG<br>ITS2 R, CGCTGCGTTCTTCATCG	300
nifH for diazotrophs	FOF1, AAAGGYGGWATCGGYAARTCCACCAC<br>FOR1, TTGTTSGCSGCRTACATSGCCATCAT	450
nifH specific for *P. triticisoli* BJ-18	*nifH* F, GCAACAGTCGGAATACGG<br>*nifH* R, TTGGGTCACGGTCATACG	217

TABLE S2. Coverage of microbial communities in the maize rhizosphere, root and shoot

Treatment	Rhizosphere			Root		Shoot	
	Bacteria	Fungi	Diazotrophs	Bacteria	Diazotrophs	Bacteria	Diazotrophs
LNE−1	0.976	**0.999**	**0.994**	**0.999**	0.989	0.999	0.989
LNE−2	0.975	**0.998**	0.995	0.999	**0.986**	0.999	**0.986**
LNE−3	0.975	0.998	0.995	0.999	0.989	0.999	0.989
LNE+1	0.977	0.998	0.995	0.999	0.990	**1.000**	0.990
LNE+2	**0.978**	0.998	0.994	0.999	0.990	0.999	0.990
LNE+3	0.978	0.999	0.995	**0.998**	**0.993**	0.999	**0.993**
MNE−1	0.976	0.998	0.995	0.999	0.987	0.999	0.987
MNE−2	0.976	0.998	**0.996**	0.999	0.988	0.999	0.988
MNE−3	0.975	0.999	0.994	0.999	0.986	0.999	0.986
MNE+1	0.977	0.998	0.996	0.999	0.987	0.999	0.987
MNE+2	0.976	0.998	0.995	0.999	0.990	0.999	0.990
MNE+3	0.975	0.999	0.995	0.999	0.989	—	0.989
HNE−1	**0.974**	0.999	0.994	0.999	0.986	0.999	0.986
HNE−2	0.975	0.998	0.995	0.999	0.988	0.999	0.988
HNE−3	0.976	0.999	0.996	0.999	0.989	0.998	0.989
HNE+1	0.975	0.998	0.995	0.999	0.988	0.999	0.988
HNE+2	0.977	0.999	0.994	0.999	0.987	**0.997**	0.987
HNE+3	0.975	0.999	0.994	0.999	0.988	0.999	0.988

LNE−: low nitrogen without inoculation; LNE+: low nitrogen with inoculation; MNE−: medium nitrogen without inoculation; MNE+: medium nitrogen with inoculation; HNE−: high nitrogen without inoculation; HNE+: high nitrogen with inoculation.

TABLE S3. Soil physicochemical properties

Treatments	pH (1:1 H_2O)	Total N ($g\ kg^{-1}$)	Available P ($mg\ kg^{-1}$)	Organic matter ($g\ kg^{-1}$)
LNE−	6.83 ± 0.12^{bc}	0.82 ± 0.02^{c}	21.73 ± 0.59^{cd}	15.59 ± 1.26^{b}
LNE+	7.07 ± 0.09^{a}	1.23 ± 0.07^{a}	29.58 ± 2.06^{a}	18.38 ± 1.32^{a}
MNE−	6.70 ± 0.06^{cd}	0.94 ± 0.03^{bc}	19.43 ± 0.27^{d}	16.04 ± 0.81^{ab}
MNE+	6.90 ± 0.06^{ab}	1.06 ± 0.08^{ab}	26.33 ± 0.47^{b}	16.76 ± 0.16^{ab}
HNE−	6.53 ± 0.09^{d}	0.94 ± 0.08^{bc}	18.00 ± 0.78^{d}	14.67 ± 0.20^{b}
HNE+	6.60 ± 0.12^{d}	0.94 ± 0.01^{bc}	23.46 ± 0.95^{b}	15.89 ± 0.36^{ab}

Values are given as mean ± SE of three independent biological replicates and values for each column not followed by the same letter different significantly according to the LSD test ($P < 0.05$); LNE−: low nitrogen without inoculation; LNE+: low nitrogen with inoculation; MNE−: medium nitrogen without inoculation; MNE+: medium nitrogen with inoculation; HNE−: high nitrogen without inoculation; HNE+: high nitrogen with inoculation; Total N: total nitrogen; Avail P: available phosphorous.

TABLE S4. Maize biomass and nutrition

	N (g kg^{-1})			P (g kg^{-1})			Dry weight (g)		
	Root	Shoot	Total	Root	Shoot	Total	Root	Shoot	Total
LNE−	23.7 ± 0.1^{c}	8.4 ± 0.5^{c}	13.0 ± 0.4^{c}	25.0 ± 1.0^{b}	12.3 ± 0.7^{c}	16.1 ± 0.7^{d}	0.147 ± 0.004^{c}	0.343 ± 0.008^{d}	0.490 ± 0.012^{d}
LNE+	34.4 ± 0.1^{b}	12.1 ± 0.7^{b}	18.9 ± 0.5^{b}	29.6 ±1.4^{a}	16.7 ± 0.8^{a}	20.6 ± 0.9^{ab}	0.184 ± 0.007^{ab}	0.423 ± 0.005a^{b}	0.607 ± 0.012^{ab}
MNE−	39.9 ± 4.5^{ab}	12.2 ± 0.3^{ab}	20.4 ± 1.5^{ab}	29.5 ± 1.9^{a}	13.1 ± 0.1^{bc}	18.0 ± 0.6^{c}	0.163 ± 0.002^{bc}	0.387 ± 0.011^{c}	0.550 ± 0.010^{c}
MNE+	42.6 ± 2.9^{a}	14.5 ± 0.5^{a}	22.8 ± 0.9^{a}	30.6 ± 0.4^{a}	16.9 ± 0.1^{a}	20.9 ± 0.2^{a}	0.182 ± 0.006^{ab}	0.436 ± 0.006^{a}	0.618 ± 0.003^{a}
HNE−	41.5 ± 2.5^{ab}	12.6 ± 0.4^{ab}	21.1 ± 1.2^{ab}	30.0 ± 0.9^{a}	14.3 ± 0.4^{b}	19.0 ± 0.4^{bc}	0.173 ± 0.005^{ab}	0.413 ± 0.007^{b}	0.587 ± 0.003^{b}
HNE+	42.8 ± 2.0^{a}	12.3 ± 1.6^{a}	21.6 ± 1.4^{a}	30.3 ± 0.9^{a}	14.6 ± 0.5^{b}	19.4 ± 0.5^{abc}	0.186 ± 0.112^{a}	0.426 ± 0.004^{ab}	0.613 ± 0.015^{ab}

Values are given as mean ± SE of three independent biological replicates and values for each column not followed by the same letter different significantly according to the LSD test ($P < 0.05$); LNE−: low nitrogen without inoculation; LNE+: low nitrogen with inoculation; MNE−: medium nitrogen without inoculation; MNE+: medium nitrogen with inoculation; HNE−: high nitrogen without inoculation; HNE+: high nitrogen with inoculation

TABLE S5. Environmental factors of Envfit in the rhizosphere microbial community

	Bacteria		Fungi		Diazotrophs	
	r^2	*p* values	r^2	*p* values	r^2	*p* values
Soil pH	0.3608	0.047	0.2459	0.117	0.5213	0.005
Soil total N	0.6756	0.001	0.4181	0.013	0.3402	0.048
Soil available P	0.7303	0.001	0.2976	0.06	0.6993	0.001
Soil OM	0.4536	0.012	0.0565	0.624	0.338	0.043
Plant TDW	0.3932	0.023	0.526	0.004	0.0792	0.54
Plant total N	0.0740	0.562	0.2052	0.199	0.215	0.144
Plant total P	0.5637	0.002	0.3832	0.030	0.2304	0.142
Soil nitrogenase	—	—	—	—	0.5859	0.003

N: nitrogen content; P: P content; OM: soil organic matter; TDW: total dry weight.

TABLE S6. Environmental factors of Envfit in the root and shoot endophytic microbial community

	Root bacteria		Root diazotrophs		Shoot bacteria		Shoot diazotrophs	
	r^2	p values	r^2	p values	r^2	p values	r^2	p values
Root DW	0.461	0.019	0.339	0.052	—	—	—	---
Root N	0.2926	0.079	0.0304	0.827	—	—	—	---
Root P	0.2893	0.08	0.125	0.401	—	—	—	---
Shoot DW	—	—	—	—	0.3188	0.071	0.4752	0.012
Shoot N	—	—	—	—	0.3694	0.044	0.4112	0.02
Shoot P	—	—	—	—	0.5358	0.01	0.5016	0.008

DW: dry weight; N: nitrogen content; P: P content.
